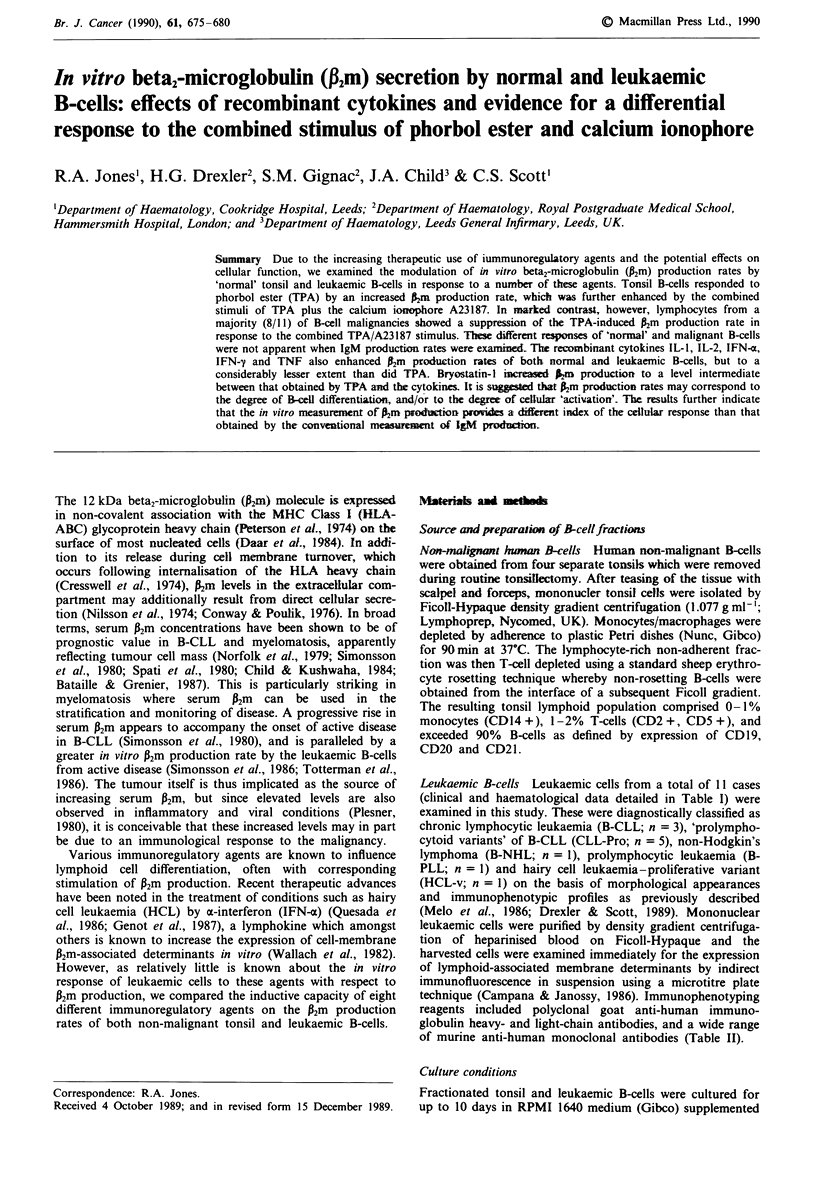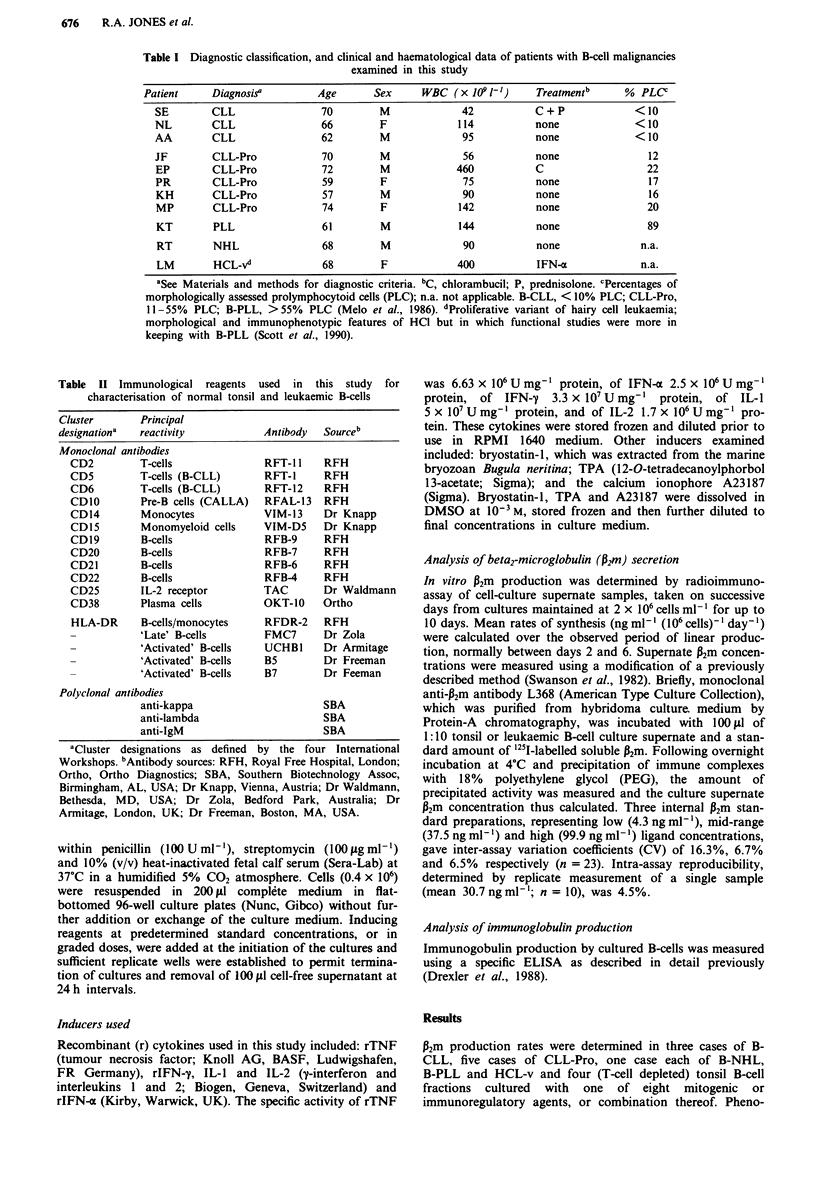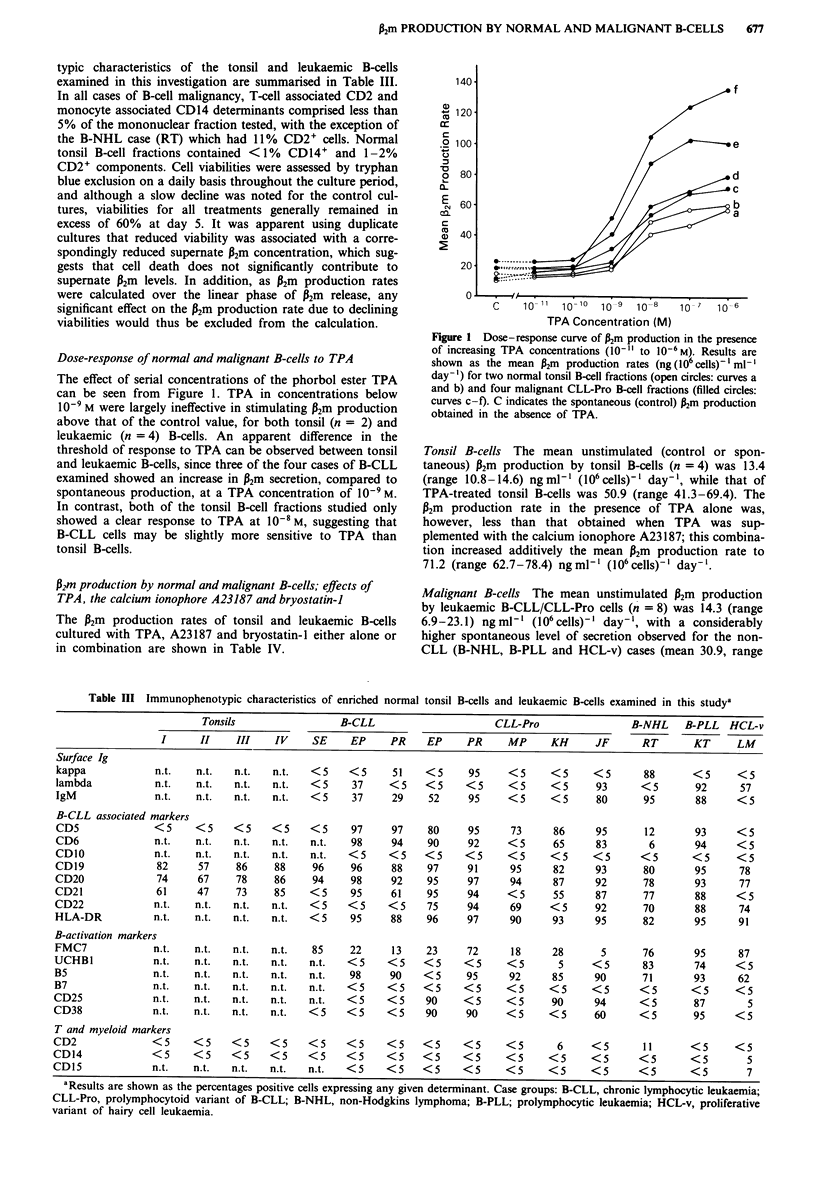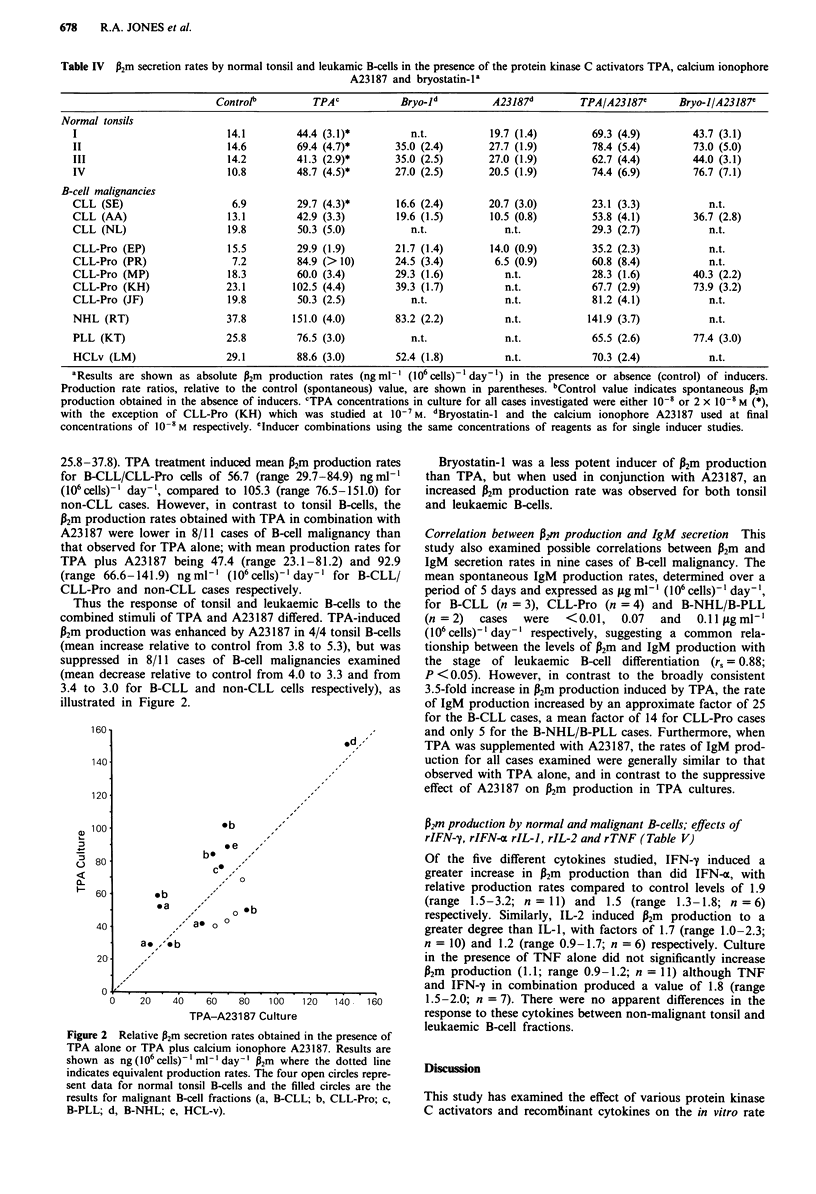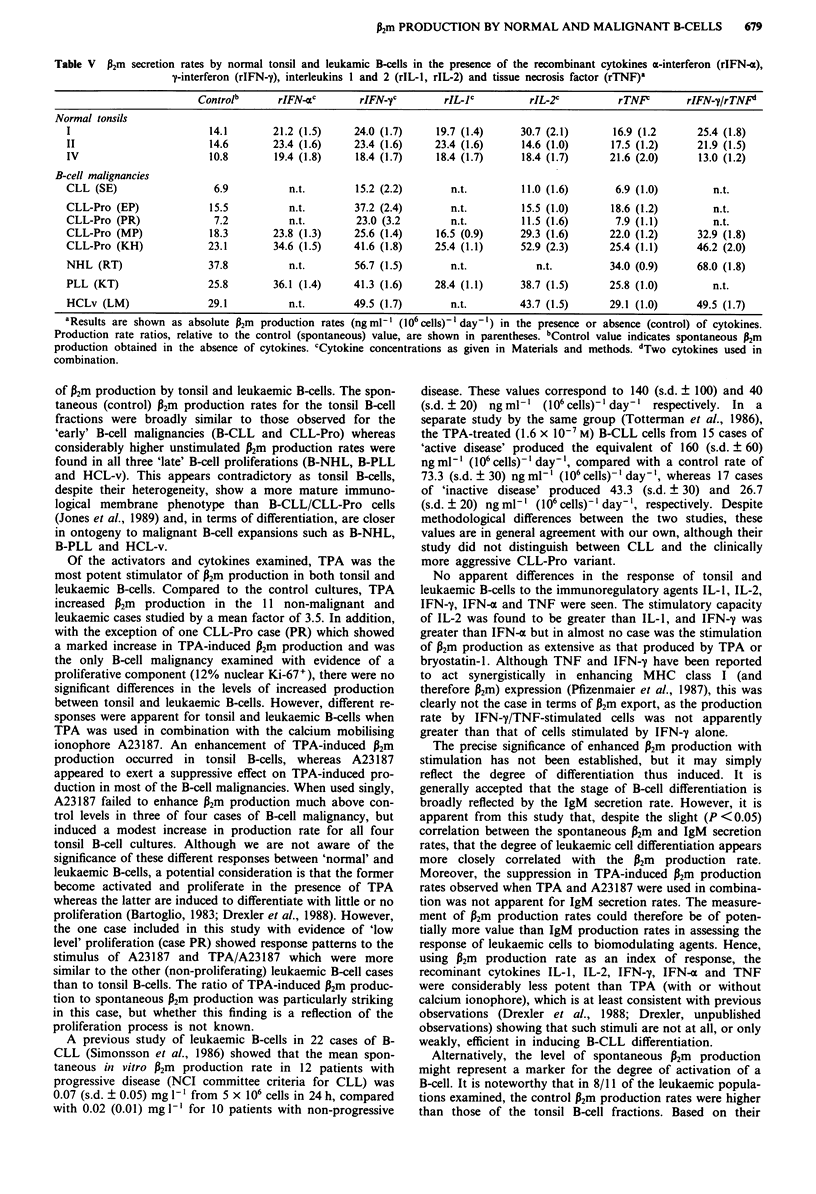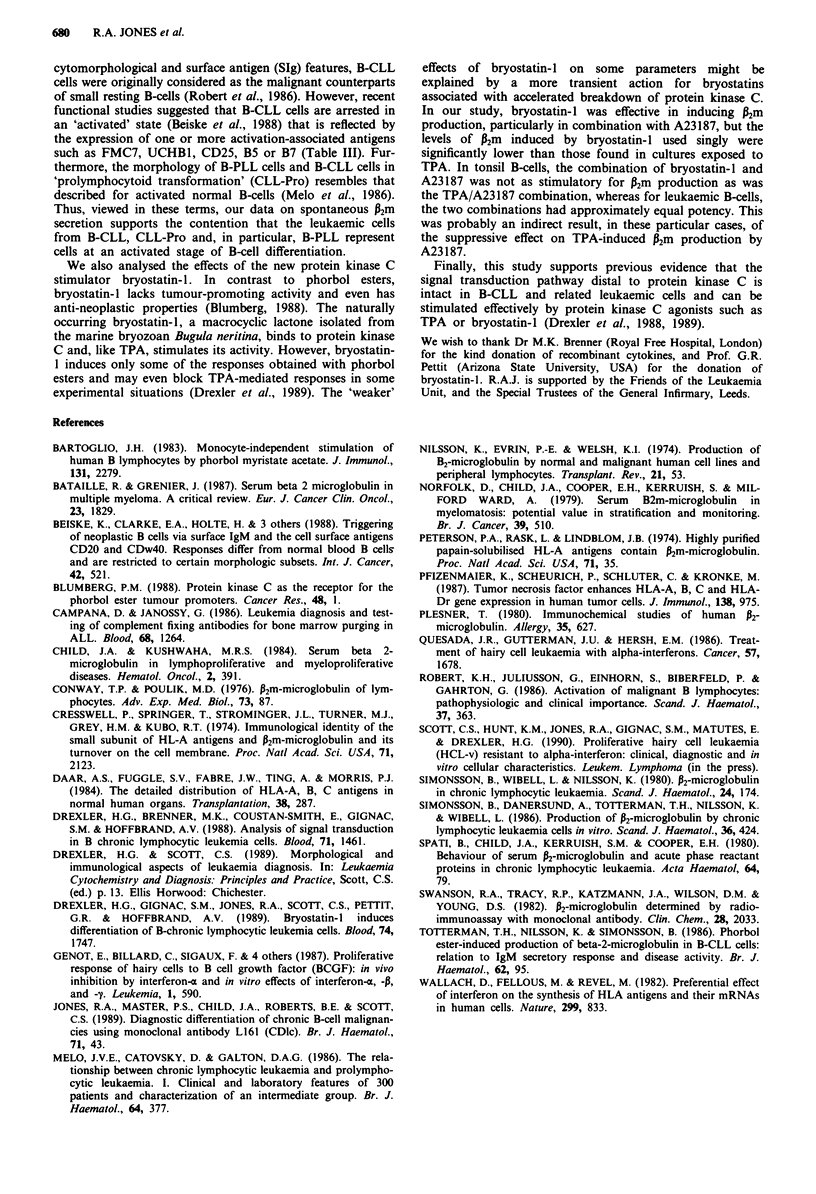# In vitro beta 2-microglobulin (beta 2m) secretion by normal and leukaemic B-cells: effects of recombinant cytokines and evidence for a differential response to the combined stimulus of phorbol ester and calcium ionophore.

**DOI:** 10.1038/bjc.1990.153

**Published:** 1990-05

**Authors:** R. A. Jones, H. G. Drexler, S. M. Gignac, J. A. Child, C. S. Scott

**Affiliations:** Department of Haematology, Cookridge Hospital, Leeds, UK.

## Abstract

Due to the increasing therapeutic use of immunoregulatory agents and the potential effects on cellular function, we examined the modulation of in vitro beta 2-microglobulin (beta 2m) production rates by 'normal' tonsil and leukaemic B-cells in response to a number of these agents. Tonsil B-cells responded to phorbol ester (TPA) by an increased beta 2m production rate, which was further enhanced by the combined stimuli of TPA plus the calcium ionophore A23187. In marked contrast, however, lymphocytes from a majority (8/11) of B-cell malignancies showed a suppression of the TPA-induced beta 2m production rate in response to the combined TPA/A23187 stimulus. These different responses of 'normal' and malignant B-cells were not apparent when IgM production rates were examined. The recombinant cytokines IL-1, IL-2, IFN-alpha, IFN-gamma and TNF also enhanced beta 2m production rates of both normal and leukaemic B-cells, but to a considerably lesser extent than did TPA. Bryostatin-1 increased beta 2m production to a level intermediate between that obtained by TPA and the cytokines. It is suggested that beta 2m production rates may correspond to the degree of B-cell differentiation, and/or to the degree of cellular 'activation'. The results further indicate that the in vitro measurement of beta 2m production provides a different index of the cellular response than that obtained by the conventional measurement of IgM production.


Br. J. Cancer (1990), 61, 675-680 © Macmillan Press Ltd., 1990

### *In vitro* beta$_2$-microglobulin ($\beta_2$m) secretion by normal and leukaemic B-cells: effects of recombinant cytokines and evidence for a differential response to the combined stimulus of phorbol ester and calcium ionophore

R.A. Jones[1], H.G. Drexler[2], S.M. Gignac[2], J.A. Child[3] & C.S. Scott[1]

[1]*Department of Haematology, Cookridge Hospital, Leeds;* [2]*Department of Haematology, Royal Postgraduate Medical School, Hammersmith Hospital, London; and* [3]*Department of Haematology, Leeds General Infirmary, Leeds, UK.*

**Summary** Due to the increasing therapeutic use of iummunoregulatory agents and the potential effects on cellular function, we examined the modulation of *in vitro* beta$_2$-microglobulin ($\beta_2$m) production rates by 'normal' tonsil and leukaemic B-cells in response to a number of these agents. Tonsil B-cells responded to phorbol ester (TPA) by an increased $\beta_2$m production rate, which was further enhanced by the combined stimuli of TPA plus the calcium ionophore A23187. In marked contrast, however, lymphocytes from a majority (8/11) of B-cell malignancies showed a suppression of the TPA-induced $\beta_2$m production rate in response to the combined TPA/A23187 stimulus. These different responses of 'normal' and malignant B-cells were not apparent when IgM production rates were examined. The recombinant cytokines IL-1, IL-2, IFN-$\alpha$, IFN-$\gamma$ and TNF also enhanced $\beta_2$m production rates of both normal and leukaemic B-cells, but to a considerably lesser extent than did TPA. Bryostatin-1 increased $\beta_2$m production to a level intermediate between that obtained by TPA and the cytokines. It is suggested that $\beta_2$m production rates may correspond to the degree of B-cell differentiation, and/or to the degree of cellular 'activation'. The results further indicate that the *in vitro* measurement of $\beta_2$m production provides a different index of the cellular response than that obtained by the conventional measurement of IgM production.

The 12 kDa beta$_2$-microglobulin ($\beta_2$m) molecule is expressed in non-covalent association with the MHC Class I (HLA-ABC) glycoprotein heavy chain (Peterson *et al.*, 1974) on the surface of most nucleated cells (Daar *et al.*, 1984). In addition to its release during cell membrane turnover, which occurs following internalisation of the HLA heavy chain (Cresswell *et al.*, 1974), $\beta_2$m levels in the extracellular compartment may additionally result from direct cellular secretion (Nilsson *et al.*, 1974; Conway & Poulik, 1976). In broad terms, serum $\beta_2$m concentrations have been shown to be of prognostic value in B-CLL and myelomatosis, apparently reflecting tumour cell mass (Norfolk *et al.*, 1979; Simonsson *et al.*, 1980; Spati *et al.*, 1980; Child & Kushwaha, 1984; Bataille & Grenier, 1987). This is particularly striking in myelomatosis where serum $\beta_2$m can be used in the stratification and monitoring of disease. A progressive rise in serum $\beta_2$m appears to accompany the onset of active disease in B-CLL (Simonsson *et al.*, 1980), and is paralleled by a greater *in vitro* $\beta_2$m production rate by the leukaemic B-cells from active disease (Simonsson *et al.*, 1986; Totterman *et al.*, 1986). The tumour itself is thus implicated as the source of increasing serum $\beta_2$m, but since elevated levels are also observed in inflammatory and viral conditions (Plesner, 1980), it is conceivable that these increased levels may in part be due to an immunological response to the malignancy.

Various immunoregulatory agents are known to influence lymphoid cell differentiation, often with corresponding stimulation of $\beta_2$m production. Recent therapeutic advances have been noted in the treatment of conditions such as hairy cell leukaemia (HCL) by $\alpha$-interferon (IFN-$\alpha$) (Quesada *et al.*, 1986; Genot *et al.*, 1987), a lymphokine which amongst others is known to increase the expression of cell-membrane $\beta_2$m-associated determinants *in vitro* (Wallach *et al.*, 1982). However, as relatively little is known about the *in vitro* response of leukaemic cells to these agents with respect to $\beta_2$m production, we compared the inductive capacity of eight different immunoregulatory agents on the $\beta_2$m production rates of both non-malignant tonsil and leukaemic B-cells.

Correspondence: R.A. Jones.
Received 4 October 1989; and in revised form 15 December 1989.

### Materials and methods

### *Source and preparation of B-cell fractions*

*Non-malignant human B-cells* Human non-malignant B-cells were obtained from four separate tonsils which were removed during routine tonsillectomy. After teasing of the tissue with scalpel and forceps, mononucler tonsil cells were isolated by Ficoll-Hypaque density gradient centrifugation (1.077 g ml$^{-1}$; Lymphoprep, Nycomed, UK). Monocytes/macrophages were depleted by adherence to plastic Petri dishes (Nunc, Gibco) for 90 min at 37°C. The lymphocyte-rich non-adherent fraction was then T-cell depleted using a standard sheep erythrocyte rosetting technique whereby non-rosetting B-cells were obtained from the interface of a subsequent Ficoll gradient. The resulting tonsil lymphoid population comprised 0-1% monocytes (CD14+), 1-2% T-cells (CD2+, CD5+), and exceeded 90% B-cells as defined by expression of CD19, CD20 and CD21.

*Leukaemic B-cells* Leukaemic cells from a total of 11 cases (clinical and haematological data detailed in Table I) were examined in this study. These were diagnostically classified as chronic lymphocytic leukaemia (B-CLL; $n$ = 3), 'prolymphocytoid variants' of B-CLL (CLL-Pro; $n$ = 5), non-Hodgkin's lymphoma (B-NHL; $n$ = 1), prolymphocytic leukaemia (B-PLL; $n$ = 1) and hairy cell leukaemia-proliferative variant (HCL-v; $n$ = 1) on the basis of morphological appearances and immunophenotypic profiles as previously described (Melo *et al.*, 1986; Drexler & Scott, 1989). Mononuclear leukaemic cells were purified by density gradient centrifugation of heparinised blood on Ficoll-Hypaque and the harvested cells were examined immediately for the expression of lymphoid-associated membrane determinants by indirect immunofluorescence in suspension using a microtitre plate technique (Campana & Janossy, 1986). Immunophenotyping reagents included polyclonal goat anti-human immunoglobulin heavy- and light-chain antibodies, and a wide range of murine anti-human monoclonal antibodies (Table II).

### *Culture conditions*

Fractionated tonsil and leukaemic B-cells were cultured for up to 10 days in RPMI 1640 medium (Gibco) supplemented

Table I Diagnostic classification, and clinical and haematological data of patients with B-cell malignancies examined in this study

| Patient | Diagnosis[a] | Age | Sex | WBC ($\times 10^9 l^{-1}$) | Treatment[b] | % PLC[c] |
|---|---|---|---|---|---|---|
| SE | CLL | 70 | M | 42 | C + P | <10 |
| NL | CLL | 66 | F | 114 | none | <10 |
| AA | CLL | 62 | M | 95 | none | <10 |
| JF | CLL-Pro | 70 | M | 56 | none | 12 |
| EP | CLL-Pro | 72 | M | 460 | C | 22 |
| PR | CLL-Pro | 59 | F | 75 | none | 17 |
| KH | CLL-Pro | 57 | M | 90 | none | 16 |
| MP | CLL-Pro | 74 | F | 142 | none | 20 |
| KT | PLL | 61 | M | 144 | none | 89 |
| RT | NHL | 68 | M | 90 | none | n.a. |
| LM | HCL-v[d] | 68 | F | 400 | IFN-α | n.a. |

[a]See Materials and methods for diagnostic criteria. [b]C, chlorambucil; P, prednisolone. [c]Percentages of morphologically assessed prolymphocytoid cells (PLC); n.a. not applicable. B-CLL, <10% PLC; CLL-Pro, 11–55% PLC; B-PLL, >55% PLC (Melo et al., 1986). [d]Proliferative variant of hairy cell leukaemia; morphological and immunophenotypic features of HCl but in which functional studies were more in keeping with B-PLL (Scott et al., 1990).

Table II Immunological reagents used in this study for characterisation of normal tonsil and leukaemic B-cells

| Cluster designation[a] | Principal reactivity | Antibody | Source[b] |
|---|---|---|---|
| *Monoclonal antibodies* | | | |
| CD2 | T-cells | RFT-11 | RFH |
| CD5 | T-cells (B-CLL) | RFT-1 | RFH |
| CD6 | T-cells (B-CLL) | RFT-12 | RFH |
| CD10 | Pre-B cells (CALLA) | RFAL-13 | RFH |
| CD14 | Monocytes | VIM-13 | Dr Knapp |
| CD15 | Monomyeloid cells | VIM-D5 | Dr Knapp |
| CD19 | B-cells | RFB-9 | RFH |
| CD20 | B-cells | RFB-7 | RFH |
| CD21 | B-cells | RFB-6 | RFH |
| CD22 | B-cells | RFB-4 | RFH |
| CD25 | IL-2 receptor | TAC | Dr Waldmann |
| CD38 | Plasma cells | OKT-10 | Ortho |
| HLA-DR | B-cells/monocytes | RFDR-2 | RFH |
| – | 'Late' B-cells | FMC7 | Dr Zola |
| – | 'Activated' B-cells | UCHB1 | Dr Armitage |
| – | 'Activated' B-cells | B5 | Dr Freeman |
| – | 'Activated' B-cells | B7 | Dr Feeman |
| *Polyclonal antibodies* | | | |
| | anti-kappa | | SBA |
| | anti-lambda | | SBA |
| | anti-IgM | | SBA |

[a]Cluster designations as defined by the four International Workshops. [b]Antibody sources: RFH, Royal Free Hospital, London; Ortho, Ortho Diagnostics; SBA, Southern Biotechnology Assoc, Birmingham, AL, USA; Dr Knapp, Vienna, Austria; Dr Waldmann, Bethesda, MD, USA; Dr Zola, Bedford Park, Australia; Dr Armitage, London, UK; Dr Freeman, Boston, MA, USA.

within penicillin (100 U $ml^{-1}$), streptomycin (100 μg $ml^{-1}$) and 10% (v/v) heat-inactivated fetal calf serum (Sera-Lab) at 37°C in a humidified 5% $CO_2$ atmosphere. Cells ($0.4 \times 10^6$) were resuspended in 200 μl complete medium in flat-bottomed 96-well culture plates (Nunc, Gibco) without further addition or exchange of the culture medium. Inducing reagents at predetermined standard concentrations, or in graded doses, were added at the initiation of the cultures and sufficient replicate wells were established to permit termination of cultures and removal of 100 μl cell-free supernatant at 24 h intervals.

### Inducers used

Recombinant (r) cytokines used in this study included: rTNF (tumour necrosis factor; Knoll AG, BASF, Ludwigshafen, FR Germany), rIFN-γ, IL-1 and IL-2 (γ-interferon and interleukins 1 and 2; Biogen, Geneva, Switzerland) and rIFN-α (Kirby, Warwick, UK). The specific activity of rTNF was $6.63 \times 10^6$ U $mg^{-1}$ protein, of IFN-α $2.5 \times 10^6$ U $mg^{-1}$ protein, of IFN-γ $3.3 \times 10^7$ U $mg^{-1}$ protein, of IL-1 $5 \times 10^7$ U $mg^{-1}$ protein, and of IL-2 $1.7 \times 10^6$ U $mg^{-1}$ protein. These cytokines were stored frozen and diluted prior to use in RPMI 1640 medium. Other inducers examined included: bryostatin-1, which was extracted from the marine bryozoan *Bugula neritina*; TPA (12-*O*-tetradecanoylphorbol 13-acetate; Sigma); and the calcium ionophore A23187 (Sigma). Bryostatin-1, TPA and A23187 were dissolved in DMSO at $10^{-3}$ M, stored frozen and then further diluted to final concentrations in culture medium.

### Analysis of beta$_2$-microglobulin ($\beta_2m$) secretion

*In vitro* $\beta_2$m production was determined by radioimmunoassay of cell-culture supernate samples, taken on successive days from cultures maintained at $2 \times 10^6$ cells $ml^{-1}$ for up to 10 days. Mean rates of synthesis (ng $ml^{-1}$ ($10^6$ cells)$^{-1}$ $day^{-1}$) were calculated over the observed period of linear production, normally between days 2 and 6. Supernate $\beta_2$m concentrations were measured using a modification of a previously described method (Swanson et al., 1982). Briefly, monoclonal anti-$\beta_2$m antibody L368 (American Type Culture Collection), which was purified from hybridoma culture. medium by Protein-A chromatography, was incubated with 100 μl of 1:10 tonsil or leukaemic B-cell culture supernate and a standard amount of $^{125}$I-labelled soluble $\beta_2$m. Following overnight incubation at 4°C and precipitation of immune complexes with 18% polyethylene glycol (PEG), the amount of precipitated activity was measured and the culture supernate $\beta_2$m concentration thus calculated. Three internal $\beta_2$m standard preparations, representing low (4.3 ng $ml^{-1}$), mid-range (37.5 ng $ml^{-1}$) and high (99.9 ng $ml^{-1}$) ligand concentrations, gave inter-assay variation coefficients (CV) of 16.3%, 6.7% and 6.5% respectively ($n = 23$). Intra-assay reproducibility, determined by replicate measurement of a single sample (mean 30.7 ng $ml^{-1}$; $n = 10$), was 4.5%.

### Analysis of immunoglobulin production

Immunogobulin production by cultured B-cells was measured using a specific ELISA as described in detail previously (Drexler et al., 1988).

### Results

$\beta_2$m production rates were determined in three cases of B-CLL, five cases of CLL-Pro, one case each of B-NHL, B-PLL and HCL-v and four (T-cell depleted) tonsil B-cell fractions cultured with one of eight mitogenic or immunoregulatory agents, or combination thereof. Phenotypic characteristics of the tonsil and leukaemic B-cells examined in this investigation are summarised in Table III. In all cases of B-cell malignancy, T-cell associated CD2 and monocyte associated CD14 determinants comprised less than 5% of the mononuclear fraction tested, with the exception of the B-NHL case (RT) which had 11% $CD2^+$ cells. Normal tonsil B-cell fractions contained <1% $CD14^+$ and 1–2% $CD2^+$ components. Cell viabilities were assessed by tryphan blue exclusion on a daily basis throughout the culture period, and although a slow decline was noted for the control cultures, viabilities for all treatments generally remained in excess of 60% at day 5. It was apparent using duplicate cultures that reduced viability was associated with a correspondingly reduced supernate $\beta_2$m concentration, which suggests that cell death does not significantly contribute to supernate $\beta_2$m levels. In addition, as $\beta_2$m production rates were calculated over the linear phase of $\beta_2$m release, any significant effect on the $\beta_2$m production rate due to declining viabilities would thus be excluded from the calculation.

### *Dose-response of normal and malignant B-cells to TPA*

The effect of serial concentrations of the phorbol ester TPA can be seen from Figure 1. TPA in concentrations below $10^{-9}$ M were largely ineffective in stimulating $\beta_2$m production above that of the control value, for both tonsil ($n = 2$) and leukaemic ($n = 4$) B-cells. An apparent difference in the threshold of response to TPA can be observed between tonsil and leukaemic B-cells, since three of the four cases of B-CLL examined showed an increase in $\beta_2$m secretion, compared to spontaneous production, at a TPA concentration of $10^{-9}$ M. In contrast, both of the tonsil B-cell fractions studied only showed a clear response to TPA at $10^{-8}$ M, suggesting that B-CLL cells may be slightly more sensitive to TPA than tonsil B-cells.

### *$\beta_2$m production by normal and malignant B-cells; effects of TPA, the calcium ionophore A23187 and bryostatin-1*

The $\beta_2$m production rates of tonsil and leukaemic B-cells cultured with TPA, A23187 and bryostatin-1 either alone or in combination are shown in Table IV.

**Figure 1** Dose–response curve of $\beta_2$m production in the presence of increasing TPA concentrations ($10^{-11}$ to $10^{-6}$ M). Results are shown as the mean $\beta_2$m production rates (ng $(10^6 \text{ cells})^{-1}$ $ml^{-1}$ $day^{-1}$) for two normal tonsil B-cell fractions (open circles: curves a and b) and four malignant CLL-Pro B-cell fractions (filled circles: curves c–f). C indicates the spontaneous (control) $\beta_2$m production obtained in the absence of TPA.

*Tonsil B-cells* The mean unstimulated (control or spontaneous) $\beta_2$m production by tonsil B-cells ($n = 4$) was 13.4 (range 10.8–14.6) ng $ml^{-1}$ $(10^6 \text{ cells})^{-1}$ $day^{-1}$, while that of TPA-treated tonsil B-cells was 50.9 (range 41.3–69.4). The $\beta_2$m production rate in the presence of TPA alone was, however, less than that obtained when TPA was supplemented with the calcium ionophore A23187; this combination increased additively the mean $\beta_2$m production rate to 71.2 (range 62.7–78.4) ng $ml^{-1}$ $(10^6 \text{ cells})^{-1}$ $day^{-1}$.

*Malignant B-cells* The mean unstimulated $\beta_2$m production by leukaemic B-CLL/CLL-Pro cells ($n = 8$) was 14.3 (range 6.9–23.1) ng $ml^{-1}$ $(10^6 \text{ cells})^{-1}$ $day^{-1}$, with a considerably higher spontaneous level of secretion observed for the non-CLL (B-NHL, B-PLL and HCL-v) cases (mean 30.9, range

**Table III** Immunophenotypic characteristics of enriched normal tonsil B-cells and leukaemic B-cells examined in this study[a]

| | *Tonsils* | | | | *B-CLL* | | | *CLL-Pro* | | | | | *B-NHL* | *B-PLL* | *HCL-v* |
|---|---|---|---|---|---|---|---|---|---|---|---|---|---|---|---|
| | *I* | *II* | *III* | *IV* | *SE* | *EP* | *PR* | *EP* | *PR* | *MP* | *KH* | *JF* | *RT* | *KT* | *LM* |
| *Surface Ig* | | | | | | | | | | | | | | | |
| kappa | n.t. | n.t. | n.t. | n.t. | <5 | <5 | 51 | <5 | 95 | <5 | <5 | <5 | 88 | <5 | <5 |
| lambda | n.t. | n.t. | n.t. | n.t. | <5 | 37 | <5 | <5 | <5 | <5 | <5 | 93 | <5 | 92 | 57 |
| IgM | n.t. | n.t. | n.t. | n.t. | <5 | 37 | 29 | 52 | 95 | <5 | <5 | 80 | 95 | 88 | <5 |
| *B-CLL associated markers* | | | | | | | | | | | | | | | |
| CD5 | <5 | <5 | <5 | <5 | <5 | 97 | 97 | 80 | 95 | 73 | 86 | 95 | 12 | 93 | <5 |
| CD6 | n.t. | n.t. | n.t. | n.t. | n.t. | 98 | 94 | 90 | 92 | <5 | 65 | 83 | 6 | 94 | <5 |
| CD10 | n.t. | n.t. | n.t. | n.t. | n.t. | <5 | <5 | <5 | <5 | <5 | <5 | <5 | <5 | <5 | <5 |
| CD19 | 82 | 57 | 86 | 88 | 96 | 96 | 88 | 97 | 91 | 95 | 82 | 93 | 80 | 95 | 78 |
| CD20 | 74 | 67 | 78 | 86 | 94 | 98 | 92 | 95 | 97 | 94 | 87 | 92 | 78 | 93 | 77 |
| CD21 | 61 | 47 | 73 | 85 | <5 | 95 | 61 | 95 | 94 | <5 | 55 | 87 | 77 | 88 | <5 |
| CD22 | n.t. | n.t. | n.t. | n.t. | <5 | <5 | <5 | 75 | 94 | 69 | <5 | 92 | 70 | 88 | 74 |
| HLA-DR | n.t. | n.t. | n.t. | n.t. | <5 | 95 | 88 | 96 | 97 | 90 | 93 | 95 | 82 | 95 | 91 |
| *B-activation markers* | | | | | | | | | | | | | | | |
| FMC7 | n.t. | n.t. | n.t. | n.t. | 85 | 22 | 13 | 23 | 72 | 18 | 28 | 5 | 76 | 95 | 87 |
| UCHB1 | n.t. | n.t. | n.t. | n.t. | n.t. | <5 | <5 | <5 | <5 | <5 | 5 | <5 | 83 | 74 | <5 |
| B5 | n.t. | n.t. | n.t. | n.t. | n.t. | 98 | 90 | <5 | 95 | 92 | 85 | 90 | 71 | 93 | 62 |
| B7 | n.t. | n.t. | n.t. | n.t. | n.t. | <5 | <5 | <5 | <5 | <5 | <5 | <5 | <5 | <5 | <5 |
| CD25 | n.t. | n.t. | n.t. | n.t. | n.t. | <5 | <5 | 90 | <5 | <5 | 90 | 94 | <5 | 87 | 5 |
| CD38 | n.t. | n.t. | n.t. | n.t. | <5 | <5 | <5 | 90 | 90 | <5 | <5 | 60 | <5 | 95 | <5 |
| *T and myeloid markers* | | | | | | | | | | | | | | | |
| CD2 | <5 | <5 | <5 | <5 | <5 | <5 | <5 | <5 | <5 | <5 | 6 | <5 | 11 | <5 | <5 |
| CD14 | <5 | <5 | <5 | <5 | <5 | <5 | <5 | <5 | <5 | <5 | <5 | <5 | <5 | <5 | 5 |
| CD15 | n.t. | n.t. | n.t. | n.t. | n.t. | <5 | <5 | <5 | <5 | <5 | <5 | <5 | <5 | <5 | 7 |

[a]Results are shown as the percentages positive cells expressing any given determinant. Case groups: B-CLL, chronic lymphocytic leukaemia; CLL-Pro, prolymphocytoid variant of B-CLL; B-NHL, non-Hodgkins lymphoma; B-PLL; prolymphocytic leukaemia; HCL-v, proliferative variant of hairy cell leukaemia.

**Table IV** $\beta_2$m secretion rates by normal tonsil and leukamic B-cells in the presence of the protein kinase C activators TPA, calcium ionophore A23187 and bryostatin-1[a]

| | *Control*[b] | *TPA*[c] | *Bryo-1*[d] | *A23187*[d] | *TPA/A23187*[e] | *Bryo-1/A23187*[e] |
|---|---|---|---|---|---|---|
| *Normal tonsils* | | | | | | |
| I | 14.1 | 44.4 (3.1)* | n.t. | 19.7 (1.4) | 69.3 (4.9) | 43.7 (3.1) |
| II | 14.6 | 69.4 (4.7)* | 35.0 (2.4) | 27.7 (1.9) | 78.4 (5.4) | 73.0 (5.0) |
| III | 14.2 | 41.3 (2.9)* | 35.0 (2.5) | 27.0 (1.9) | 62.7 (4.4) | 44.0 (3.1) |
| IV | 10.8 | 48.7 (4.5)* | 27.0 (2.5) | 20.5 (1.9) | 74.4 (6.9) | 76.7 (7.1) |
| *B-cell malignancies* | | | | | | |
| CLL (SE) | 6.9 | 29.7 (4.3)* | 16.6 (2.4) | 20.7 (3.0) | 23.1 (3.3) | n.t. |
| CLL (AA) | 13.1 | 42.9 (3.3) | 19.6 (1.5) | 10.5 (0.8) | 53.8 (4.1) | 36.7 (2.8) |
| CLL (NL) | 19.8 | 50.3 (5.0) | n.t. | n.t. | 29.3 (2.7) | n.t. |
| CLL-Pro (EP) | 15.5 | 29.9 (1.9) | 21.7 (1.4) | 14.0 (0.9) | 35.2 (2.3) | n.t. |
| CLL-Pro (PR) | 7.2 | 84.9 (>10) | 24.5 (3.4) | 6.5 (0.9) | 60.8 (8.4) | n.t. |
| CLL-Pro (MP) | 18.3 | 60.0 (3.4) | 29.3 (1.6) | n.t. | 28.3 (1.6) | 40.3 (2.2) |
| CLL-Pro (KH) | 23.1 | 102.5 (4.4) | 39.3 (1.7) | n.t. | 67.7 (2.9) | 73.9 (3.2) |
| CLL-Pro (JF) | 19.8 | 50.3 (2.5) | n.t. | n.t. | 81.2 (4.1) | n.t. |
| NHL (RT) | 37.8 | 151.0 (4.0) | 83.2 (2.2) | n.t. | 141.9 (3.7) | n.t. |
| PLL (KT) | 25.8 | 76.5 (3.0) | n.t. | n.t. | 65.5 (2.6) | 77.4 (3.0) |
| HCLv (LM) | 29.1 | 88.6 (3.0) | 52.4 (1.8) | n.t. | 70.3 (2.4) | n.t. |

[a]Results are shown as absolute $\beta_2$m production rates (ng ml$^{-1}$ (10$^6$ cells)$^{-1}$ day$^{-1}$) in the presence or absence (control) of inducers. Production rate ratios, relative to the control (spontaneous) value, are shown in parentheses. [b]Control value indicates spontaneous $\beta_2$m production obtained in the absence of inducers. [c]TPA concentrations in culture for all cases investigated were either $10^{-8}$ or $2 \times 10^{-8}$ M (*), with the exception of CLL-Pro (KH) which was studied at $10^{-7}$ M. [d]Bryostatin-1 and the calcium ionophore A23187 used at final concentrations of $10^{-8}$ M respectively. [e]Inducer combinations using the same concentrations of reagents as for single inducer studies.

25.8–37.8). TPA treatment induced mean $\beta_2$m production rates for B-CLL/CLL-Pro cells of 56.7 (range 29.7–84.9) ng ml$^{-1}$ (10$^6$ cells)$^{-1}$ day$^{-1}$, compared to 105.3 (range 76.5–151.0) for non-CLL cases. However, in contrast to tonsil B-cells, the $\beta_2$m production rates obtained with TPA in combination with A23187 were lower in 8/11 cases of B-cell malignancy than that observed for TPA alone; with mean production rates for TPA plus A23187 being 47.4 (range 23.1–81.2) and 92.9 (range 66.6–141.9) ng ml$^{-1}$ (10$^6$ cells)$^{-1}$ day$^{-1}$ for B-CLL/CLL-Pro and non-CLL cases respectively.

Thus the response of tonsil and leukaemic B-cells to the combined stimuli of TPA and A23187 differed. TPA-induced $\beta_2$m production was enhanced by A23187 in 4/4 tonsil B-cells (mean increase relative to control from 3.8 to 5.3), but was suppressed in 8/11 cases of B-cell malignancies examined (mean decrease relative to control from 4.0 to 3.3 and from 3.4 to 3.0 for B-CLL and non-CLL cells respectively), as illustrated in Figure 2.

**Figure 2** Relative $\beta_2$m secretion rates obtained in the presence of TPA alone or TPA plus calcium ionophore A23187. Results are shown as ng (10$^6$ cells)$^{-1}$ ml$^{-1}$ day$^{-1}$ $\beta_2$m where the dotted line indicates equivalent production rates. The four open circles represent data for normal tonsil B-cells and the filled circles are the results for malignant B-cell fractions (a, B-CLL; b, CLL-Pro; c, B-PLL; d, B-NHL; e, HCL-v).

Bryostatin-1 was a less potent inducer of $\beta_2$m production than TPA, but when used in conjunction with A23187, an increased $\beta_2$m production rate was observed for both tonsil and leukaemic B-cells.

*Correlation between $\beta_2$m production and IgM secretion* This study also examined possible correlations between $\beta_2$m and IgM secretion rates in nine cases of B-cell malignancy. The mean spontaneous IgM production rates, determined over a period of 5 days and expressed as µg ml$^{-1}$ (10$^6$ cells)$^{-1}$ day$^{-1}$, for B-CLL ($n = 3$), CLL-Pro ($n = 4$) and B-NHL/B-PLL ($n = 2$) cases were <0.01, 0.07 and 0.11 µg ml$^{-1}$ (10$^6$ cells)$^{-1}$ day$^{-1}$ respectively, suggesting a common relationship between the levels of $\beta_2$m and IgM production with the stage of leukaemic B-cell differentiation ($r_s = 0.88$; $P < 0.05$). However, in contrast to the broadly consistent 3.5-fold increase in $\beta_2$m production induced by TPA, the rate of IgM production increased by an approximate factor of 25 for the B-CLL cases, a mean factor of 14 for CLL-Pro cases and only 5 for the B-NHL/B-PLL cases. Furthermore, when TPA was supplemented with A23187, the rates of IgM production for all cases examined were generally similar to that observed with TPA alone, and in contrast to the suppressive effect of A23187 on $\beta_2$m production in TPA cultures.

*$\beta_2$m production by normal and malignant B-cells; effects of rIFN-γ, rIFN-α rIL-1, rIL-2 and rTNF (Table V)*

Of the five different cytokines studied, IFN-γ induced a greater increase in $\beta_2$m production than did IFN-α, with relative production rates compared to control levels of 1.9 (range 1.5–3.2; $n = 11$) and 1.5 (range 1.3–1.8; $n = 6$) respectively. Similarly, IL-2 induced $\beta_2$m production to a greater degree than IL-1, with factors of 1.7 (range 1.0–2.3; $n = 10$) and 1.2 (range 0.9–1.7; $n = 6$) respectively. Culture in the presence of TNF alone did not significantly increase $\beta_2$m production (1.1; range 0.9–1.2; $n = 11$) although TNF and IFN-γ in combination produced a value of 1.8 (range 1.5–2.0; $n = 7$). There were no apparent differences in the response to these cytokines between non-malignant tonsil and leukaemic B-cell fractions.

### Discussion

This study has examined the effect of various protein kinase C activators and recombinant cytokines on the *in vitro* rate

**Table V** $\beta_2$m secretion rates by normal tonsil and leukamic B-cells in the presence of the recombinant cytokines α-interferon (rIFN-α), γ-interferon (rIFN-γ), interleukins 1 and 2 (rIL-1, rIL-2) and tissue necrosis factor (rTNF)[a]

| | *Control*[b] | *rIFN-α*[c] | *rIFN-γ*[c] | *rIL-1*[c] | *rIL-2*[c] | *rTNF*[c] | *rIFN-γ/rTNF*[d] |
|---|---|---|---|---|---|---|---|
| *Normal tonsils* | | | | | | | |
| I | 14.1 | 21.2 (1.5) | 24.0 (1.7) | 19.7 (1.4) | 30.7 (2.1) | 16.9 (1.2 | 25.4 (1.8) |
| II | 14.6 | 23.4 (1.6) | 23.4 (1.6) | 23.4 (1.6) | 14.6 (1.0) | 17.5 (1.2) | 21.9 (1.5) |
| IV | 10.8 | 19.4 (1.8) | 18.4 (1.7) | 18.4 (1.7) | 18.4 (1.7) | 21.6 (2.0) | 13.0 (1.2) |
| *B-cell malignancies* | | | | | | | |
| CLL (SE) | 6.9 | n.t. | 15.2 (2.2) | n.t. | 11.0 (1.6) | 6.9 (1.0) | n.t. |
| CLL-Pro (EP) | 15.5 | n.t. | 37.2 (2.4) | n.t. | 15.5 (1.0) | 18.6 (1.2) | n.t. |
| CLL-Pro (PR) | 7.2 | n.t. | 23.0 (3.2 | n.t. | 11.5 (1.6) | 7.9 (1.1) | n.t. |
| CLL-Pro (MP) | 18.3 | 23.8 (1.3) | 25.6 (1.4) | 16.5 (0.9) | 29.3 (1.6) | 22.0 (1.2) | 32.9 (1.8) |
| CLL-Pro (KH) | 23.1 | 34.6 (1.5) | 41.6 (1.8) | 25.4 (1.1) | 52.9 (2.3) | 25.4 (1.1) | 46.2 (2.0) |
| NHL (RT) | 37.8 | n.t. | 56.7 (1.5) | n.t. | n.t. | 34.0 (0.9) | 68.0 (1.8) |
| PLL (KT) | 25.8 | 36.1 (1.4) | 41.3 (1.6) | 28.4 (1.1) | 38.7 (1.5) | 25.8 (1.0) | n.t. |
| HCLv (LM) | 29.1 | n.t. | 49.5 (1.7) | n.t. | 43.7 (1.5) | 29.1 (1.0) | 49.5 (1.7) |

[a]Results are shown as absolute $\beta_2$m production rates (ng ml$^{-1}$ ($10^6$ cells)$^{-1}$ day$^{-1}$) in the presence or absence (control) of cytokines. Production rate ratios, relative to the control (spontaneous) value, are shown in parentheses. [b]Control value indicates spontaneous $\beta_2$m production obtained in the absence of cytokines. [c]Cytokine concentrations as given in Materials and methods. [d]Two cytokines used in combination.

of $\beta_2$m production by tonsil and leukaemic B-cells. The spontaneous (control) $\beta_2$m production rates for the tonsil B-cell fractions were broadly similar to those observed for the 'early' B-cell malignancies (B-CLL and CLL-Pro) whereas considerably higher unstimulated $\beta_2$m production rates were found in all three 'late' B-cell proliferations (B-NHL, B-PLL and HCL-v). This appears contradictory as tonsil B-cells, despite their heterogeneity, show a more mature immunological membrane phenotype than B-CLL/CLL-Pro cells (Jones *et al.*, 1989) and, in terms of differentiation, are closer in ontogeny to malignant B-cell expansions such as B-NHL, B-PLL and HCL-v.

Of the activators and cytokines examined, TPA was the most potent stimulator of $\beta_2$m production in both tonsil and leukaemic B-cells. Compared to the control cultures, TPA increased $\beta_2$m production in the 11 non-malignant and leukaemic cases studied by a mean factor of 3.5. In addition, with the exception of one CLL-Pro case (PR) which showed a marked increase in TPA-induced $\beta_2$m production and was the only B-cell malignancy examined with evidence of a proliferative component (12% nuclear Ki-67$^+$), there were no significant differences in the levels of increased production between tonsil and leukaemic B-cells. However, different responses were apparent for tonsil and leukaemic B-cells when TPA was used in combination with the calcium mobilising ionophore A23187. An enhancement of TPA-induced $\beta_2$m production occurred in tonsil B-cells, whereas A23187 appeared to exert a suppressive effect on TPA-induced production in most of the B-cell malignancies. When used singly, A23187 failed to enhance $\beta_2$m production much above control levels in three of four cases of B-cell malignancy, but induced a modest increase in production rate for all four tonsil B-cell cultures. Although we are not aware of the significance of these different responses between 'normal' and leukaemic B-cells, a potential consideration is that the former become activated and proliferate in the presence of TPA whereas the latter are induced to differentiate with little or no proliferation (Bartoglio, 1983; Drexler *et al.*, 1988). However, the one case included in this study with evidence of 'low level' proliferation (case PR) showed response patterns to the stimulus of A23187 and TPA/A23187 which were more similar to the other (non-proliferating) leukaemic B-cell cases than to tonsil B-cells. The ratio of TPA-induced $\beta_2$m production to spontaneous $\beta_2$m production was particularly striking in this case, but whether this finding is a reflection of the proliferation process is not known.

A previous study of leukaemic B-cells in 22 cases of B-CLL (Simonsson *et al.*, 1986) showed that the mean spontaneous *in vitro* $\beta_2$m production rate in 12 patients with progressive disease (NCI committee criteria for CLL) was 0.07 (s.d. ± 0.05) mg l$^{-1}$ from $5 \times 10^6$ cells in 24 h, compared with 0.02 (0.01) mg l$^{-1}$ for 10 patients with non-progressive disease. These values correspond to 140 (s.d. ± 100) and 40 (s.d. ± 20) ng ml$^{-1}$ ($10^6$ cells)$^{-1}$ day$^{-1}$ respectively. In a separate study by the same group (Totterman *et al.*, 1986), the TPA-treated ($1.6 \times 10^{-7}$ M) B-CLL cells from 15 cases of 'active disease' produced the equivalent of 160 (s.d. ± 60) ng ml$^{-1}$ ($10^6$ cells)$^{-1}$ day$^{-1}$, compared with a control rate of 73.3 (s.d. ± 30) ng ml$^{-1}$ ($10^6$ cells)$^{-1}$ day$^{-1}$, whereas 17 cases of 'inactive disease' produced 43.3 (s.d. ± 30) and 26.7 (s.d. ± 20) ng ml$^{-1}$ ($10^6$ cells)$^{-1}$ day$^{-1}$, respectively. Despite methodological differences between the two studies, these values are in general agreement with our own, although their study did not distinguish between CLL and the clinically more aggressive CLL-Pro variant.

No apparent differences in the response of tonsil and leukaemic B-cells to the immunoregulatory agents IL-1, IL-2, IFN-γ, IFN-α and TNF were seen. The stimulatory capacity of IL-2 was found to be greater than IL-1, and IFN-γ was greater than IFN-α but in almost no case was the stimulation of $\beta_2$m production as extensive as that produced by TPA or bryostatin-1. Although TNF and IFN-γ have been reported to act synergistically in enhancing MHC class I (and therefore $\beta_2$m) expression (Pfizenmaier *et al.*, 1987), this was clearly not the case in terms of $\beta_2$m export, as the production rate by IFN-γ/TNF-stimulated cells was not apparently greater than that of cells stimulated by IFN-γ alone.

The precise significance of enhanced $\beta_2$m production with stimulation has not been established, but it may simply reflect the degree of differentiation thus induced. It is generally accepted that the stage of B-cell differentiation is broadly reflected by the IgM secretion rate. However, it is apparent from this study that, despite the slight ($P < 0.05$) correlation between the spontaneous $\beta_2$m and IgM secretion rates, that the degree of leukaemic cell differentiation appears more closely correlated with the $\beta_2$m production rate. Moreover, the suppression in TPA-induced $\beta_2$m production rates observed when TPA and A23187 were used in combination was not apparent for IgM secretion rates. The measurement of $\beta_2$m production rates could therefore be of potentially more value than IgM production rates in assessing the response of leukaemic cells to biomodulating agents. Hence, using $\beta_2$m production rate as an index of response, the recominant cytokines IL-1, IL-2, IFN-γ, IFN-α and TNF were considerably less potent than TPA (with or without calcium ionophore), which is at least consistent with previous observations (Drexler *et al.*, 1988; Drexler, unpublished observations) showing that such stimuli are not at all, or only weakly, efficient in inducing B-CLL differentiation.

Alternatively, the level of spontaneous $\beta_2$m production might represent a marker for the degree of activation of a B-cell. It is noteworthy that in 8/11 of the leukaemic populations examined, the control $\beta_2$m production rates were higher than those of the tonsil B-cell fractions. Based on their cytomorphological and surface antigen (SIg) features, B-CLL cells were originally considered as the malignant counterparts of small resting B-cells (Robert *et al.*, 1986). However, recent functional studies suggested that B-CLL cells are arrested in an 'activated' state (Beiske *et al.*, 1988) that is reflected by the expression of one or more activation-associated antigens such as FMC7, UCHB1, CD25, B5 or B7 (Table III). Furthermore, the morphology of B-PLL cells and B-CLL cells in 'prolymphocytoid transformation' (CLL-Pro) resembles that described for activated normal B-cells (Melo *et al.*, 1986). Thus, viewed in these terms, our data on spontaneous $\beta_2$m secretion supports the contention that the leukaemic cells from B-CLL, CLL-Pro and, in particular, B-PLL represent cells at an activated stage of B-cell differentiation.

We also analysed the effects of the new protein kinase C stimulator bryostatin-1. In contrast to phorbol esters, bryostatin-1 lacks tumour-promoting activity and even has anti-neoplastic properties (Blumberg, 1988). The naturally occurring bryostatin-1, a macrocyclic lactone isolated from the marine bryozoan *Bugula neritina*, binds to protein kinase C and, like TPA, stimulates its activity. However, bryostatin-1 induces only some of the responses obtained with phorbol esters and may even block TPA-mediated responses in some experimental situations (Drexler *et al.*, 1989). The 'weaker' effects of bryostatin-1 on some parameters might be explained by a more transient action for bryostatins associated with accelerated breakdown of protein kinase C. In our study, bryostatin-1 was effective in inducing $\beta_2$m production, particularly in combination with A23187, but the levels of $\beta_2$m induced by bryostatin-1 used singly were significantly lower than those found in cultures exposed to TPA. In tonsil B-cells, the combination of bryostatin-1 and A23187 was not as stimulatory for $\beta_2$m production as was the TPA/A23187 combination, whereas for leukaemic B-cells, the two combinations had approximately equal potency. This was probably an indirect result, in these particular cases, of the suppressive effect on TPA-induced $\beta_2$m production by A23187.

Finally, this study supports previous evidence that the signal transduction pathway distal to protein kinase C is intact in B-CLL and related leukaemic cells and can be stimulated effectively by protein kinase C agonists such as TPA or bryostatin-1 (Drexler *et al.*, 1988, 1989).

We wish to thank Dr M.K. Brenner (Royal Free Hospital, London) for the kind donation of recombinant cytokines, and Prof. G.R. Pettit (Arizona State University, USA) for the donation of bryostatin-1. R.A.J. is supported by the Friends of the Leukaemia Unit, and the Special Trustees of the General Infirmary, Leeds.

### References

BARTOGLIO, J.H. (1983). Monocyte-independent stimulation of human B lymphocytes by phorbol myristate acetate. *J. Immunol.*, **131**, 2279.

BATAILLE, R. & GRENIER, J. (1987). Serum beta 2 microglobulin in multiple myeloma. A critical review. *Eur. J. Cancer Clin. Oncol.*, **23**, 1829.

BEISKE, K., CLARKE, E.A., HOLTE, H. & 3 others (1988). Triggering of neoplastic B cells via surface IgM and the cell surface antigens CD20 and CDw40. Responses differ from normal blood B cells and are restricted to certain morphologic subsets. *Int. J. Cancer*, **42**, 521.

BLUMBERG, P.M. (1988). Protein kinase C as the receptor for the phorbol ester tumour promoters. *Cancer Res.*, **48**, 1.

CAMPANA, D. & JANOSSY, G. (1986). Leukemia diagnosis and testing of complement fixing antibodies for bone marrow purging in ALL. *Blood*, **68**, 1264.

CHILD, J.A. & KUSHWAHA, M.R.S. (1984). Serum beta 2-microglobulin in lymphoproliferative and myeloproliferative diseases. *Hematol. Oncol.*, **2**, 391.

CONWAY, T.P. & POULIK, M.D. (1976). $\beta_2$m-microglobulin of lymphocytes. *Adv. Exp. Med. Biol.*, **73**, 87.

CRESSWELL, P., SPRINGER, T., STROMINGER, J.L., TURNER, M.J., GREY, H.M. & KUBO, R.T. (1974). Immunological identity of the small subunit of HL-A antigens and $\beta_2$m-microglobulin and its turnover on the cell membrane. *Proc. Natl Acad. Sci. USA*, **71**, 2123.

DAAR, A.S., FUGGLE, S.V., FABRE, J.W., TING, A. & MORRIS, P.J. (1984). The detailed distribution of HLA-A, B, C antigens in normal human organs. *Transplantation*, **38**, 287.

DREXLER, H.G., BRENNER, M.K., COUSTAN-SMITH, E., GIGNAC, S.M. & HOFFBRAND, A.V. (1988). Analysis of signal transduction in B chronic lymphocytic leukemia cells. *Blood*, **71**, 1461.

DREXLER, H.G. & SCOTT, C.S. (1989). Morphological and immunological aspects of leukaemia diagnosis. In: *Leukaemia Cytochemistry and Diagnosis: Principles and Practice*, Scott, C.S. (ed.) p. 13. Ellis Horwood: Chichester.

DREXLER, H.G., GIGNAC, S.M., JONES, R.A., SCOTT, C.S., PETTIT, G.R. & HOFFBRAND, A.V. (1989). Bryostatin-1 induces differentiation of B-chronic lymphocytic leukemia cells. *Blood*, **74**, 1747.

GENOT, E., BILLARD, C., SIGAUX, F. & 4 others (1987). Proliferative response of hairy cells to B cell growth factor (BCGF): *in vivo* inhibition by interferon-$\alpha$ and *in vitro* effects of interferon-$\alpha$, -$\beta$, and -$\gamma$. *Leukemia*, **1**, 590.

JONES, R.A., MASTER, P.S., CHILD, J.A., ROBERTS, B.E. & SCOTT, C.S. (1989). Diagnostic differentiation of chronic B-cell malignancies using monoclonal antibody L161 (CDlc). *Br. J. Haematol.*, **71**, 43.

MELO, J.V.E., CATOVSKY, D. & GALTON, D.A.G. (1986). The relationship between chronic lymphocytic leukaemia and prolymphocytic leukaemia. I. Clinical and laboratory features of 300 patients and characterization of an intermediate group. *Br. J. Haematol.*, **64**, 377.

NILSSON, K., EVRIN, P.-E. & WELSH, K.I. (1974). Production of $B_2$-microglobulin by normal and malignant human cell lines and peripheral lymphocytes. *Transplant. Rev.*, **21**, 53.

NORFOLK, D., CHILD, J.A., COOPER, E.H., KERRUISH, S. & MILFORD WARD, A. (1979). Serum B2m-microglobulin in myelomatosis: potential value in stratification and monitoring. *Br. J. Cancer*, **39**, 510.

PETERSON, P.A., RASK, L. & LINDBLOM, J.B. (1974). Highly purified papain-solubilised HL-A antigens contain $\beta_2$m-microglobulin. *Proc. Natl Acad. Sci. USA*, **71**, 35.

PFIZENMAIER, K., SCHEURICH, P., SCHLUTER, C. & KRONKE, M. (1987). Tumor necrosis factor enhances HLA-A, B, C and HLA-Dr gene expression in human tumor cells. *J. Immunol.*, **138**, 975.

PLESNER, T. (1980). Immunochemical studies of human $\beta_2$-microglobulin. *Allergy*, **35**, 627.

QUESADA, J.R., GUTTERMAN, J.U. & HERSH, E.M. (1986). Treatment of hairy cell leukaemia with alpha-interferons. *Cancer*, **57**, 1678.

ROBERT, K.H., JULIUSSON, G., EINHORN, S., BIBERFELD, P. & GAHRTON, G. (1986). Activation of malignant B lymphocytes: pathophysiologic and clinical importance. *Scand. J. Haematol.*, **37**, 363.

SCOTT, C.S., HUNT, K.M., JONES, R.A., GIGNAC, S.M., MATUTES, E. & DREXLER, H.G. (1990). Proliferative hairy cell leukaemia (HCL-v) resistant to alpha-interferon: clinical, diagnostic and *in vitro* cellular characteristics. *Leukem. Lymphoma* (in the press).

SIMONSSON, B., WIBELL, L. & NILSSON, K. (1980). $\beta_2$-microglobulin in chronic lymphocytic leukaemia. *Scand. J. Haematol.*, **24**, 174.

SIMONSSON, B., DANERSUND, A., TOTTERMAN, T.H., NILSSON, K. & WIBELL, L. (1986). Production of $\beta_2$-microglobulin by chronic lymphocytic leukaemia cells *in vitro*. *Scand. J. Haematol.*, **36**, 424.

SPATI, B., CHILD, J.A., KERRUISH, S.M. & COOPER, E.H. (1980). Behaviour of serum $\beta_2$-microglobulin and acute phase reactant proteins in chronic lymphocytic leukaemia. *Acta Haematol*, **64**, 79.

SWANSON, R.A., TRACY, R.P., KATZMANN, J.A., WILSON, D.M. & YOUNG, D.S. (1982). $\beta_2$-microglobulin determined by radioimmunoassay with monoclonal antibody. *Clin. Chem.*, **28**, 2033.

TOTTERMAN, T.H., NILSSON, K. & SIMONSSON, B. (1986). Phorbol ester-induced production of beta-2-microglobulin in B-CLL cells: relation to IgM secretory response and disease activity. *Br. J. Haematol.*, **62**, 95.

WALLACH, D., FELLOUS, M. & REVEL, M. (1982). Preferential effect of interferon on the synthesis of HLA antigens and their mRNAs in human cells. *Nature*, **299**, 833.